# Therapeutic effects of atorvastatin and ezetimibe compared with double-dose atorvastatin in very elderly patients with acute coronary syndrome

**DOI:** 10.18632/oncotarget.15078

**Published:** 2017-02-03

**Authors:** Zhi Liu, Hengjian Hao, Chunlin Yin, Yanyan Chu, Jing Li, Dong Xu

**Affiliations:** ^1^ Division of Cardiology, Xuanwu Hospital Capital Medical University, Beijing, China

**Keywords:** elderly, acute coronary syndrome, atorvastatin, ezetimibe, prognosis

## Abstract

Objective Compared the effect of atorvastatin 10 mg combined ezetimibe 10 mg therapy with atorvastatin 20 mg on the long-term outcomes in very elderly patients with acute coronary syndrome.

Methods A total of 230 octogenarian patients with acute coronary syndrome underwent coronary angiography were randomized to combined therapy group (atorvastatin 10 mg/d and ezetimibe 10 mg/d, n=114) or double-dose atorvastatin group (atorvastatin 20mg/d, n=116). The primary end point was one-year incidence of major adverse cardiovascular events (including cardiac death, spontaneous myocardial infarction, unplanned revascularization).

Result At the end of one year, the percentage of patients with low-density lipoprotein cholesterol level decreased more than 30% or 50% were comparable between the two groups (93.5% vs. 90.1%, p= 0.36; 54.6% vs. 49.6%, p= 0.45). The rate of major adverse cardiovascular events in combined therapy group was similar with double-dose atorvastatin group (23.2% vs. 19.8%, p=0.55). In COX regression model, the risk of major adverse cardiovascular events in combined group isn’t significantly higher than double-dose atorvastatin group (HR [95% CI] 1.12 [0.51 to 2.55], p = 0.74). The patients whose alanine aminotransferase increasing more than upper normal limit in combined group was lower than double-dose atorvastatin group (2.8% vs. 9.0%, p = 0.05).

Conclusions For very elderly patients with acute coronary syndrome, atorvastatin combining ezetimibe induced similar long-term outcomes compared with double-dose atorvastatin but with less liver dysfunction.

Percutaneous coronary intervention (PCI) is an important method in the treatment of coronary artery disease. However, culprit lesion revascularization after acute coronary syndrome (ACS) reduces the relative risk of mid- to-long-term cardiovascular events by only 20% [1], nearly about 50% events are likely to arise from sites remote to the original culprit lesion [2]. Low-density lipoprotein cholesterol (LDL) level is strongly associated with the increased incident of coronary artery disease (CAD) and many guidelines recommend intensive lipid-lowing therapy for patients at very high CAD risk [3, 4]. Some trials suggested long-term high-dose statins therapy could make greater plaque regression and can improve the prognosis of ACS patients [5, 6]. On the other hand, clinicians often worry about the potential harm of high-dose statin when consider its’ benefit especially for elderly. Geriatric patients are at risk for drug side effects because of polypharmacy, alterations in the rate of first pass and phase I metabolism, and decreased capacity of carrier proteins such as albumin [7]. Ezetimibe is one kind of lipid-lowering drug known as cholesterol absorption inhibitors which has different metabolic pathway with statins [8]. One trial indicated ezetimibe (10mg/day) plus low-dose atorvastatin (10mg/day) had a similar reduction of LDL-C with high-dose atorvastatin (40mg/day) [9]. There were few randomized clinical trials (RCTs) of statin or other hypocholesterolemic medication included persons older than 80 years [10].This trial was designed to test the efficacy and safety of ezetimibe (10mg/day) plus atorvastatin 10mg/day for octogenarian patients with ACS in one year follow-up compared with atorvastatin 20 mg/day.

## METHODS

### Study population and design

This was a randomized controlled trial in Xuanwu Hospital of Capital Medical University from June 2012 to December 2014. The protocol had been previously approved by the institutional review board and all patients provided informed consent. Clinical enrollment criteria were: 1) acute coronary syndrome (ACS) patients confirmed by coronary angiography; 2) age between 80 and 90 years old. Exclusion criteria were: chronic high-dose statins therapy (atorvastatin> 10mg/day), referral to coronary artery bypass surgery (CABG), abnormal liver enzymes (alanine aminotransferase (ALT) or aspartate aminotransferase (AST)> 40U/L); renal failure with serum creatinine> 2 mg/dl, muscle disease or refused the trial. Eligible patients were randomized 1:1 to combined therapy group (atorvastatin 10 mg/d and ezetimibe 10 mg/d) or double-dose atorvastatin group (atorvastatin 20mg/d). Specifically, a randomization list was provided by the sponsor before the beginning of the study using SPSS Statistics version 20.0.0 computer software. Block randomization was used with a block size equal to 2, each block containing one patient who was assigned combined therapy and one patient who was assigned double-dose atorvastatin.

All PCI were performed with standard technique and only drug-eluting stents were used. Patients’ blood samples were collected in hospital to measure creatine kinase myocardial band (CK-MB), troponin-I (TNI), creatine (CK), ALT, AST, creatinine, low-density lipoprotein cholesterol (LDL-C), high sensitive C-reactive protein (hsCRP) levels; further measurements were asked to perform at 3, 6, 12 month after discharge. Patients were followed up with telephone calls at the end of one year. The primary end point was one-year incidence of major adverse cardiovascular events (MACE, including cardiac death, spontaneous myocardial infarction, unplanned revascularization). Spontaneous myocardial infarction was defined as: detection of rise of cardiac biomarkers (preferably troponin) with at least one value above the 99^th^ percentile of the upper reference limit together with evidence of myocardial ischemia with at least one of the following: Symptoms of ischemia; electrocardiogram change indicative of new ischemia(new ST-T changes, or new left bundle branch block); development of pathological Q waves in electrocardiogram; imaging evidence of new loss of viable myocardium or new regional wall motion abnormality [11]. An unplanned revascularization was defined as a revascularization procedure that was not planned at the first angiography of the admission.

### Statistical analysis

Statistical analysis was performed using JMP version 10.0.0 (SAS Institute, Inc. Cary, NC). Continuous variables were presented as mean ± standard deviation, or median values for those with a skewed distribution. Discrete variables were summarized as absolute numbers and percentages. Inter-group comparisons were tested with independent t-test for normally distributed continuous variables and Wilcoxon rank sum test for skewed continuous variables, and Pearson's χ^2^ test was used for categorical variables. Cumulative event rates were estimated with the Kaplan-Meier method and differences were tested with a log-rank test. The Cox proportional hazard regression models were used to explore the association between the survival of patients and clinical factors. We built multivariable models by adjusting for age, gender, body mass index (BMI), current smoking, prior CAD (prior MI and PCI), hypertension, diabetes mellitus, hyperlipidemia, ST-segment elevation myocardial infarction (STEMI), PCI. All significance tests were two-tailed. Statistical significance was defined as a P value less than 0.05.

## RESULT

### Study population

From June 2012 to December 2014, a total of 264 patients (accounted for 7.1% of all the ACS patients) fulfilling the enrollment criteria and 34 patients were initially excluded: 13 patients had chronic high-dose statin therapy, 4 patients refused the trail, 10 patients had abnormal liver function, and 7 patients had chronic renal failure. At last, there were 230 patients were randomized to combined therapy group (atorvastatin 10 mg/d and ezetimibe 10mg/day for one year, n =114) or double-dose atorvastatin group (20 mg/day for one year, n = 116). Clinical features of the study patients were showed in Table 1. Age, gender, coronary artery disease history, hypertension, lipid level, liver function, renal function and medical therapy were comparable between the two groups. The two groups’ angiography and PCI characteristics were similar. There were 52 patients in combined group (45.6%) and 50 patients in double-dose group (43.1%) implanted drug-eluting stents.

**Table 1:** Clinical baseline and coronary angiography characters

Variable	Combined therapy (*n* =114 )	Double-dose (*n* = 116)	*P* value
Male, n (%)	60 (52.6)	59 (50.9)	0.79
Age, years	84.2±2.9	84.0±1.8	0.73
BMI, kg/m^2^	25.6±3.5	25.4±3.9	0.72
STEMI, n (%)	46(40.4)	43(37.1)	0.61
HT, n (%)	81 (71.1)	80 (69.0)	0.73
DM, n (%)	46(40.4)	42(36.2)	0.52
Current smokers, n (%)	13 (11.4)	16 (13.8)	0.59
Previous MI, n (%)	22 (19.3)	17 (14.7)	0.35
Previous PCI, n (%)	16 (14.0)	12 (10.3)	0.39
Previous stroke, n (%)	14 (12.3)	16 (13.8)	0.73
LVEF, (%)	61.7±11.4	61.2±9.9	0.79
Aspirin, n (%)	98(86.0)	92(79.3)	0.18
clopidogrel, n (%)	85 (74.6)	78 (67.2)	0.22
ACEI/ARB, n (%)	89 (78.1)	85(73.3)	0.40
β-blocker, n (%)	76 (66.7)	74 (63.8)	0.65
Alt, IU/L	22.7±15.5	22.1±17.6	0.88
CRE, umol/L	85.3±22.3	79.6±25.2	0.44
hsCRP, mg/L	3.1±1.6	3.2±1.8	0.42
LDL-C, mmol/L	2.2±0.6	2.3±0.8	0.15
HDL-C, mmol/L	1.2±0.3	1.3±0.3	0.12
TG, mmol/L	1.5±1.0	1.6±1.5	0.32
Left main intervention, n (%)	15 (13.2)	15 (12.9)	0.33
Single-vessel disease, n (%)	40 (35.1)	48 (41.4)	0.33
No. of vessel diseases, n	2.1±1.1	1.9±0.9	0.87
contrast media, ml	105.8±66.9	103.2±67.6	0.61
PCI patients, n (%)	52 (45.6)	50 (43.1)	0.70
In PCI patients			
Stent number (n)	1.7±0.7	1.9±1.0	0.14
Stent diameter, mm	3.0±0.5	3.0±0.4	0.72
Total stent length, mm	43.8±20.7	48.8±28.3	0.16

Age, gender, hypertension, lipid level, liver function, renal function, medical therapy and angiography characteristics were similar between the two groups.

Abbreviations: BMI, body mass index; STEMI, ST-segment elevation myocardial infarction; LVEF, left ventricular ejection fraction; ACEI, Angiotensin-converting enzyme inhibitors; ARB, angiotensin receptor blocker; ALT, alanine aminotransferase; CRE, creatinine; TG, triglyceride; HDL-C, high-density lipoprotein cholesterol; LDL-C, low-density lipoprotein cholesterol; CK, creatine kinase; hsCRP, high sensitive C-reactive protein; PCI, percutaneous coronary intervention.

### The prognosis in the two groups

**Figure 1:**
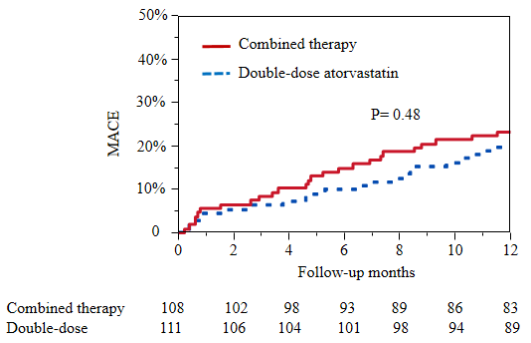
Unadjusted Kaplan-Meier curves for MACE of the two groups in one-year follow-up The event rate of MACE in combined therapy group was similar with double-dose atorvastatin group. MACE, major adverse cardiovascular events, including cardiac death, spontaneous myocardial infarction, unplanned revascularization.

There were 6 patients in combined therapy group and 5 in double-dose atorvastatin group lost to follow-up. In the first 30 days, MACE rates were not significantly different between combined therapy group and double-dose atorvastatin group (5.6% vs. 4.5%, p = 0.72). The similar situation appeared in the PCI patients of the two groups (5.8% vs. 4.3, p= 0.73). At the end of one year, the rates of MACE, cardiac death, MI, unplanned revascularization, and stroke in combined therapy group were all comparable with double-dose atorvastatin group (Table 2). Figure 1 showed the MACE rate of combined therapy group was similar to double-dose atorvastatin group in the one-year follow-up (p = 0.48). Relative to patients with double-dose atorvastatin, patients with combined therapy had no significantly higher risks for MACE in COX model after adjusting for age, gender, BMI, current smoking, prior CAD, hypertension, diabetes mellitus, hyperlipidemia, STEMI, PCI (HR [95% CI] 1.12 [0.51 to 2.55], p = 0.74). In the one-year, there were 33 patients (22 patients with PCI) in combined therapy group and 26 patients (18 patients with PCI) in double-dose atorvastatin group performed coronary arteriography because of chest pain. In the PCI subgroup, there were a non-significant increase of MACE in the combined therapy group compared with double-dose atorvastatin group (26.9% vs. 19.2%, p = 0.36).

**Table 2:** The outcomes of the two groups in one year

Variable	Combined therapy (*n* =108 )	Double-dose (*n* =111)	*P* value
MACE, n (%)	25(23.2)	22 (19.8)	0.55
Cardiac death, n (%)	5 (4.6)	5 (4.6)	0.96
Spontaneous MI, n (%)	10 (9.3)	11 (9.9)	0.87
Unplanned revascularization, n (%)	10 (9.3)	6 (5.4)	0.27
Stroke, n (%)	13 (12.0)	11 (9.9)	0.48

The event rates were comparable between the two groups in one year.

Abbreviations: MACE, major adverse coronary events; MI, myocardial infarction.

### Biochemistry data and safety

LDL-C level decreased 38.4% in the combined therapy group and 34.7% in the double-dose atorvastatin group in the first three months (p = 0.36), which was 43.6% and 40.1% at the end of one year (p = 0.41). The number of patients with LDL-C decreased more than 30% (moderate intensity lipid-lowering therapy) was 81 in the combined therapy group and 74 in double-dose atorvastatin group (75.0% vs. 66.7%, p = 0.18) at the end of three months, which were 101 and 100 at the end of one year (93.5% vs. 90.1%, p= 0.36). The number of patients with LDL-C decreased more than 50% (high intensity lipid-lowering therapy) was 47 in the combined therapy group and 44 in double-dose atorvastatin group (43.5% vs. 39.6%, p = 0.56), which were 59 and 55 at the end of one year (54.6% vs. 49.6%, p= 0.45). At the end of three months, the hsCRP level of the combined treatment group was higher than that of the double-dose group (2.5±1.4 vs. 2.1±1.2 mg/L, p= 0.034). This difference became indistinct at the end of 6 months and one year. There were 3 patients in combined therapy group and 10 patients in double-dose atorvastatin group whose alanine aminotransferase (ALT) more than upper normal limit (2.8% vs. 9.0%, p = 0.05), 1 patient in combined therapy group and 3 patients in double-dose atorvastatin group whose ALT more than 3-fold (0.93% vs. 2.7%, p = 0.33). There was no patients whose ALT more than 5-fold upper normal limit. After descending transaminase treatment, they all recovered and continue previous therapy. There was no confirmed adverse drug reactions to muscle in both groups. There was one new cancer case in each group. The number of patients with new-onset diabetes was 3 in combined therapy group and 5 in double-dose atorvastatin group (2.8% vs. 4.5%, p = 0.50). These biochemical data were shown in Table 3.

**Table 3:** Biochemical data of the two groups in follow-up

	months		6 months		12 months	
	Combined therapy	Double-dose	Combined therapy	Double-dose	Combined therapy	Double-dose
ALT, IU/L	28.2±18.9	34.1±17.2	29.5±11.8	35.3±12.7	29.2±14.9	36.1±14.3
CRE, umol/L	78.7±22.3	77.4±23.1	77.8±25.2	76.9±21.2	76.2±16.3	75.2±22.5
TG, mmol/L	1.3±0.9	1.1±0.5	1.3±0.7	1.2±0.9	1.4±0.9	1.3±0.9
HDL-C, mmol/L	1.0±0.3	1.1±0.4	1.1±0.2	1.2±0.3	1.2±0.4	1.2±0.3
LDL-C, mmol/L	1.4±0.5	1.5±0.6	1.3±0.5	1.4±0.6	1.2±0.6	1.4±0.7
CK, U/L	78.9±36.2	85.6±38.9	83.4±42.5	91.2±41.2	82.0±36.2	90.3±49.2
hsCRP, mg/L	2.5±1.3	2.1±1.2*	1.8±1.1	1.7±1.1	1.2±1.0	1.1±0.8

* hsCRP, compared with combined therapy group, *p* < 0.05.

In fact, both LDL-C and hsCRP decreased faster in double-dose atorvastatin group than combined therapy in the first three months.

Abbreviations: ALT, alanine aminotransferase; CRE, creatinine; TG, triglyceride; HDL-C, high-density lipoprotein cholesterol; LDL-C, low-density lipoprotein cholesterol; CK, creatine kinase; hsCRP, high sensitive C-reactive protein.

## DISCUSSION

The current study indicated that atorvastatin combining ezetimibe induced similar long-term outcomes compared with double-dose atorvastatin but with less liver dysfunction for very elderly patients with ACS.

Cholesterol levels decrease in elderly may because of the development of cholesterol metabolism, malnutrition, frailty or chronic diseases [12–14]. Comparison with younger patients, the absolute effects of cholesterol level on CAD mortality rates are much greater in older patients [10]. Among 80 to 89 years old patients, the annual CAD mortality rate increased 10-fold more compared with 40 to 49 years olds for each 1-mmol/L increase in total cholesterol levels [15]. A meta-analysis reported that each 1-mmol/L reduction in LDL-C decreases the annual rate of arteriosclerotic cardiovascular disease (ASCVD) by more than one-fifth and all-cause mortality by 10% regardless of age [16]. ACC/AHA guideline supports starting statin treatment in patients aged 75 to 82 years with clinical ASCVD [17].

However, elderly patients may be more prone to adverse effects of statins [18, 19]. Muscle effects range from pain without elevated serum creatinine kinase levels to rhabdomyolysis [20]. Other considerations include increases in liver transaminase levels, which usually resolve after dose reduction, or discontinuation of the drug, or may also normalize spontaneously [21]. In recent years, it has been observed that the use of statins increases the risk of type 2 diabetes [22, 23]. In fact, in 2012 the European Medicines Agency (EMA) published guidelines related to an increased risk of diabetes associated with statin therapy [24]. This effect is dose-dependent and has a clear relationship with age [25–29]. Age older than 75 to 80 years is often regarded as a risk factor for adverse effects^17^. It has been reported that 47% of patients > 75 are on >5 drugs [30]. On the other hand, a study of > 950,000 patient records from US databases showed that 83% of patients with dyslipidemia used a CYP3A4-metabolised statin and that, of these, 25%-30% also received a CYP3A4 inhibitor [31]. This suggests that elderly patients treated with statins have a particularly high risk of developing drug-drug interactions especially with high dose. The ACC/AHA guideline recommends a moderate intensity (but not a high-intensity) statin treatment for ASCVD patients older than 75 years [17].

Ezetimibe can inhibit the absorption of intestinal cholesterol by act on Niemann-Pick C1-Like 1 (NPC1L1), which is a polytopic transmembrane protein localized at the apical membrane of enterocytes and the canalicular membrane of hepatocytes. NPC1L1 is a sterol transporter to mediate intestinal cholesterol absorption and counterbalances hepatobiliary cholesterol excretion [32]. Ezetimibe alone played the same protection against a moderate atherosclerotic lesion, which was associated with lowering serum cholesterol, decreasing circulating inflammatory cytokines, and inhibiting macrophage accumulation in the lesions [33]. When added to statin therapy, ezetimibe resulted in incremental lowering of LDL-C levels and improved cardiovascular outcomes [34, 35]. But some other trails didn't support this conclusion [36, 37]. This trail showed that the low dose atorvastatin combined with ezetimibe introduce similar clinical outcomes of elderly ACS patients compared with double dose of atorvastatin, but the incidence of adverse effect was reduced.

A possible benefit from high-dose atorvastatin for elderly ACS patients with PCI was observed, especially reduction in in-stent restenosis or thrombosis. Statins can accelerate vascular healing process after DES implantation, which maybe profit from reducing endothelial inflammatory response, improving endothelial dysfunction and having antioxidant effects [38].

### Limitations

However, this was a single-center study, and the sample size was small. If the number of patients enlarged, some difference maybe become significant. The medication adherence was not assessed except atorvastatin and ezetimibe. A larger RCT is needed to determine the best dose of lipid-lowering agents in secondary prevention for elderly ACS patients.

## References

[1] Cannon CP, Weintraub WS, Demopoulos LA, Vicari R, Frey MJ, Lakkis N, Neumann FJ, Robertson DH, DeLucca PT, DiBattiste PM, Gibson CM, Braunwald E (2001). TACTICS (Treat Angina with Aggrastat and Determine Cost of Therapy with an Invasive or Conservative Strategy)--Thrombolysis in Myocardial Infarction 18 Investigators. Comparison of early invasive and conservative strategies in patients with unstable coronary syndromes treated with the glycoprotein IIb/IIIa inhibitor tirofiban. N Engl J Med.

[2] Stone GW, Maehara A, Lansky AJ, de Bruyne B, Cristea E, Mintz GS, Mehran R, McPherson J, Farhat N, Marso SP, Parise H, Templin B, White R, Zhang Z, Investigators Serruys PW (2011). PROSPECT A prospective natural-history study of coronary atherosclerosis. N Engl J Med.

[3] Perk J, De Backer G, Gohlke H, Graham I, Reiner Z, Verschuren M, Albus C, Benlian P, Boysen G, Cifkova R, Deaton C, Ebrahim S, Fisher M, European Association for Cardiovascular Prevention & Rehabilitation (EACPR), ESC Committee for Practice Guidelines (CPG) (2012). European Guidelines on cardiovascular disease prevention in clinical practice (version 2012). The Fifth Joint Task Force of the European Society of Cardiology and Other Societies on Cardiovascular Disease Prevention in Clinical Practice (constituted by representatives of nine societies and by invited experts). Eur Heart J.

[4] Stone NJ, Robinson JG, Lichtenstein AH, CN Bairey Merz, Blum CB, Eckel RH, Goldberg AC, Gordon D, Levy D, Lloyd-Jones DM, McBride P, Schwartz JS, Shero ST (2014). American College of Cardiology/American Heart Association Task Force on Practice Guidelines. 2013 ACC/AHA guideline on the treatment of blood cholesterol to reduce atherosclerotic cardiovascular risk in adults: a report of the American College of Cardiology/American Heart Association Task Force on Practice Guidelines. J Am Coll Cardiol.

[5] Puri R, Nissen SE, Shao M, Ballantyne CM, Barter PJ, Chapman MJ, Erbel R, Libby P, Raichlen JS, Uno K, Kataoka Y, Nicholls SJ (2014). Antiatherosclerotic effects of long-term maximally intensive statin therapy after acute coronary syndrome: insights from Study of Coronary Atheroma by Intravascular Ultrasound: Effect of Rosuvastatin Versus Atorvastatin. Arterioscler Thromb Vasc Biol.

[6] Murphy SA, Cannon CP, Wiviott SD, McCabe CH, Braunwald E (2009). Reduction in recurrent cardiovascular events with intensive lipid-lowering statin therapy compared with moderate lipid-lowering statin therapy after acute coronary syndromes from the PROVE IT-TIMI 22 (Pravastatin or Atorvastatin Evaluation and Infection Therapy-Thrombolysis In Myocardial Infarction 22) trial. J Am Coll Cardiol.

[7] Van Dam J, Zeldis JB (1990). Hepatic diseases in the elderly. Gastroenterol Clin North Am.

[8] Davidson MH (2003). Ezetimibe: a novel option for lowering cholesterol. Expert Rev Cardiovasc Ther.

[9] Piorkowski M, Fischer S, Stellbaum C, Jaster M, Martus P, Morguet AJ, Schultheiss HP, Rauch U (2007). Treatment with ezetimibe plus low-dose atorvastatin compared with higher-dose atorvastatin alone: is sufficient cholesterol-lowering enough to inhibit platelets?. J Am Coll Cardiol.

[10] Strandberg TE, Kolehmainen L, Vuorio A (2014). Evaluation and treatment of older patients with hypercholesterolemia: a clinical review. JAMA.

[11] Thygesen K, Alpert JS, Jaffe AS, Simoons ML, Chaitman BR, White HD (2012). Joint ESC/ACCF/AHA/WHF Task Force for Universal Definition of Myocardial Infarction. Third universal definition of myocardial infarction. J Am Coll Cardiol.

[12] Afilalo J, Alexander KP, Mack MJ, Maurer MS, Green P, Allen LA, Popma JJ, Ferrucci L, Forman DE (2014). Frailty assessment in the cardiovascular care of older adults. J Am Coll Cardiol.

[13] Fontana L, Addante F, Copetti M, Paroni G, Fontana A, Sancarlo D, Pellegrini F, Ferrucci L, Pilotto A (2013). Identification of a metabolic signature for multidimensional impairment and mortality risk in hospitalized older patients. Aging Cell.

[14] Tilvis RS, Valvanne JN, Strandberg TE, Miettinen TA (2011). Prognostic significance of serum cholesterol, lathosterol, and sitosterol in old age; a 17-year population study. Ann Med.

[15] Collaboration Prospective Studies, Lewington S, Whitlock G, Clarke R, Sherliker P, Emberson J, Halsey J, Qizilbash N, Peto R, Collins R (2007). Prospective Studies Collaboration. Blood cholesterol and vascular mortality by age, sex, and blood pressure: a meta-analysis of individual data from 61 prospective studies with 55,000 vascular deaths. Lancet.

[16] Baigent C, Blackwell L, Emberson J, Holland LE, Reith C, Bhala N, Peto R, Barnes EH, Keech A, Simes J, Collins R, Cholesterol Treatment Trialists’ CTT Collaboration (2010). Cholesterol Treatment Trialists’ (CTT) Collaboration. Efficacy and safety of more intensive lowering of LDL cholesterol: a meta-analysis of data from 170,000 participants in 26 randomised trials. Lancet.

[17] Stone NJ, Robinson JG, Lichtenstein AH, CN Bairey Merz, Blum CB, Eckel RH, Goldberg AC, Gordon D, Levy D, Lloyd-Jones DM, McBride P, Schwartz JS, Shero ST (2014). 2013 ACC/AHA guideline on the treatment of blood cholesterol to reduce atherosclerotic cardiovascular risk in adults: a report of the American College of Cardiology/American Heart Association Task Force on Practice Guidelines. Circulation.

[18] Jukema JW, Cannon CP, de Craen AJM, Westendorp RGJ, Trompet S (2012). The controversies of statin therapy: weighing the evidence. J Am Coll Cardiol.

[19] Macedo AF, Taylor FC, Casas JP, Adler A, Prieto-Merino D, Ebrahim S (2014). Unintended effects of statins from observational studies in the general population: systematic review and meta-analysis. BMC Med.

[20] Bruckert E, Hayem G, Dejager S, Yau C, Bégaud B (2005). Mild to moderate muscular symptoms with high-dosage statin therapy in hyperlipidemic patients-the PRIMO study. Cardiovasc Drugs Ther.

[21] Argo CK, Loria P, Caldwell SH, Lonardo A (2008). Statins in liver disease: a molehill, an iceberg, or neither?. Hepatology.

[22] Sattar N, Preiss D, Murray HM, Welsh P, Buckley BM, de Craen AJ, Seshasai SR, McMurray JJ, Freeman DJ, Jukema JW, Macfarlane PW, Packard CJ, Stott DJ (2010). Statins and risk of incident diabetes: a collaborative meta-analysis of randomized statin trials. Lancet.

[23] Ridker PM, Pradhan A, MacFadyen JG, Libby P, Glynn RJ (2012). Cardiovascular benefits and diabetes risks of statin therapy in primary prevention: an analysis from the JUPITER trial. Lancet.

[24] Pharmacovigilance Working Party (PhVWP) (2011). plenary meeting, 2012. European Medicines Agency Website.

[25] Waters DD, Ho JE, DeMicco DA, Breazna A, Arsenault BJ, Wun CC, Kastelein JJ, Colhoun H, Barter P (2011). Predictors of new-onset diabetes in patients treated with atorvastatin: results from 3 large randomized clinical trials. J Am Coll Cardiol.

[26] Preiss D, Seshasai SR, Welsh P, Murphy SA, Ho JE, Waters DD, DeMicco DA, Barter P, Cannon CP, Sabatine MS, Braunwald E, Kastelein JJ, de Lemos JA (2011). Risk of incident diabetes with intensive-dose compared with moderate-dose statin therapy: a meta-analysis. JAMA.

[27] Carter AA, Gomes T, Camacho X, Juurlink DN, Shah BR, Mamdani MM (2013). Risk of incident diabetes among patient treated with statins: population based study. BMJ.

[28] Zaharan NL, Williams D, Bennett K (2013). Statins and risk of treated incident diabetes in a primary care population. Br J Clin Pharmacol.

[29] Dormuth CR, Filion KB, Paterson JM, James MT, Teare GF, Raymond CB, Rahme E, Tamim H, Lipscombe L (2014). Canadian Network for Observational Drug Effect Studies Investigators. Higher potency statins and the risk of new diabetes: multicentre, observational study of administrative databases. BMJ.

[30] Wilmot KA, Khan A, Krishnan S, Eapen DJ, Sperling L (2015). Statins in the elderly: a patient-focused approach. Clin Cardiol.

[31] Ming EE, Davidson MH, Gandhi SK, Marotti M, Miles CG, Ke X, McKenney JM (2008). Concomitant use of statins and CYP3A4 inhibitors in administrative claims and electronic medical records databases. J Clin Lipidol.

[32] Jia L, Betters JL, Yu L (2011). Niemann-pick C1-like 1 (NPC1L1) protein in intestinal and hepatic cholesterol transport. Annu Rev Physiol.

[33] Tie Chunmiao, Gao Kanglu, Zhang Na, Zhang Songzhao, Shen Jiali, Xie Xiaojie, Wang Jian-an (2015). Ezetimibe attenuates atherosclerosis associated with lipid reduction and inflammation inhibition. PLoS One.

[34] Cannon CP, Blazing MA, Giugliano RP, McCagg A, White JA, Theroux P, Darius H, Lewis BS, Ophuis TO, Jukema JW, De Ferrari GM, Ruzyllo W, De Lucca P (2015). Ezetimibe Added to Statin Therapy after Acute Coronary Syndromes. N Engl J Med.

[35] Baigent C, Landray MJ, Reith C, Emberson J, Wheeler DC, Tomson C, Wanner C, Krane V, Cass A, Craig J, Neal B, Jiang L, Hooi LS (2011). The effects of lowering LDL cholesterol with simvastatin plus ezetimibe in patients with chronic kidney disease (Study of Heart and Renal Protection): a randomised placebo-controlled trial. Lancet.

[36] Kastelein JJ, Akdim F, Stroes ES, Zwinderman AH, Bots ML, Stalenhoef AF, Visseren FL, Sijbrands EJ, Trip MD, Stein EA, Gaudet D, Duivenvoorden R, Veltri EP (2008). Simvastatin with or without ezetimibe in familial hypercholesterolemia. N Engl J Med.

[37] Fleg JL, Mete M, Howard BV, Umans JG, Roman MJ, Ratner RE, Silverman A, Galloway JM, Henderson JA, Weir MR, Wilson C, Stylianou M, Howard WJ (2008). Effect of statins alone versus statins plus ezetimibe on carotid atherosclerosis in type 2 diabetes: the SANDS (Stop Atherosclerosis in Native Diabetics Study) trial. J Am Coll Cardiol.

[38] Stoekenbroek RM, Boekholdt SM, Fayyad R, Laskey R, Tikkanen MJ, Pedersen TR, Hovingh GK (2015). Incremental decrease in end points through aggressive lipid lowering study group. High-dose atorvastatin is superior to moderate-dose simvastatin in preventing peripheral arterial disease. Heart.

